# Reducing functional dysconnectivity in people with schizophrenia spectrum disorders

**DOI:** 10.1192/bjo.2025.10892

**Published:** 2025-11-10

**Authors:** Stephan Wunderlich, Daniel Keeser, Johanna Spaeth, Deniz Yilmaz, Isabel Maurus, Cagatay Alici, Andrea Schmitt, Peter Falkai, Sophia Stoecklein, Lukas Roell

**Affiliations:** Department of Radiology, LMU University Hospital, Ludwig Maximilian University Munich, Munich, Germany; Department of Psychiatry and Psychotherapy, LMU University Hospital, Ludwig Maximilian University Munich, Munich, Germany; NeuroImaging Core Unit Munich (NICUM), LMU University Hospital, Ludwig Maximilian University Munich, Munich, Germany; Laboratory of Neuroscience (LIM27), Institute of Psychiatry, University of Sao Paulo, São Paulo, Brazil; DZPG (German Center for Mental Health), Partner Site Munich/Augsburg, Munich, Germany; Max Planck Institute for Psychiatry, Munich, Germany; Department of Neurology, Klinikum Nuremberg, Nuremberg, Germany

**Keywords:** Psychotic disorders/schizophrenia, neuroimaging, general adult psychiatry, network analysis, sports and exercise psychiatry

## Abstract

**Background:**

The dysconnection hypothesis of schizophrenia posits that widespread synaptic inefficiencies lead to altered macroscale brain connectivity, contributing to symptom severity and cognitive deficits in individuals with schizophrenia spectrum disorders (SSD). Emerging evidence suggests that physical exercise may help to ameliorate these connectivity abnormalities and associated clinical impairments.

**Aims:**

This study investigated whether reductions in functional dysconnectivity following exercise therapy were associated with clinical improvements in individuals with SSD. In addition, it explored the genetic underpinnings of these changes using imaging transcriptomics.

**Method:**

Using data from the ESPRIT C3 trial, we analysed 23 SSD patients (seven female) undergoing aerobic exercise or flexibility, strengthening and balance training over 6 months. Functional dysconnectivity, assessed at baseline and post-intervention relative to a healthy reference sample (*n* = 200), was evaluated at the whole-brain, network and regional levels. Linear mixed effect models and voxel-wise Pearson’s correlations were used to assess exercise-induced changes and clinical relevance.

**Results:**

Functional dysconnectivity significantly decreased (*d* = −2.73, *P* < 0.001), and this decrease was primarily linked to enhanced oligodendrocyte-related gene expression. Reductions in the default-mode network were correlated with improved global functioning, whereas changes in insular regions were associated with symptom severity and functioning. Dysconnectivity reductions in somatomotor and frontoparietal networks were correlated with total symptom improvements, and changes in language-related regions (e.g. Broca’s area) were linked to cognitive benefits.

**Conclusions:**

Our findings support the role of oligodendrocyte pathology in SSD and suggest that targeting dysconnectivity in the default-mode, salience and language networks may enhance global functioning, symptom severity and cognitive impairments.

The dysconnection hypothesis of schizophrenia claims that psychosis is characterised by impaired synaptic efficacy throughout the brain, leading to widespread macroscale deteriorations in intrinsic and extrinsic connectivity related to schizophrenia-typical positive, negative and cognitive symptoms.^1^ Accordingly, several large-scale neuroimaging studies have demonstrated multiple aberrant functional connectivity patterns in people with schizophrenia spectrum disorders (SSD) compared with healthy controls.^2,3^ In particular, functional dysconnectivity within and between core brain networks, such as the default-mode, salience, frontoparietal, somatomotor, limbic, visual, subcortical and dorsal attention networks, reflects a stable transdiagnostic neural marker in psychiatry as assessed by functional magnetic resonance imaging (fMRI).^3–5^ Concerning SSD-specific alterations, several particular dysconnectivity patterns have been proposed. For instance, specific seeds within the default-mode, salience, frontoparietal and limbic networks show functional dysconnectivity in individuals with SSD but not in those with other psychiatric conditions.^3^ Moreover, certain functional disturbances within numerous essential circuits, such as the cortico-striato-pallido-thalamo-cortical circuit,^3,6^ have been frequently reported in patients with SSD. Another central epicentre of continuous neural decline throughout the disease course of psychosis is the hippocampal formation,^7^ whose connections with the prefrontal cortex are reported to be disturbed in SSD.^8^ As part of the cortico-striato-pallido-thalamo-cortical loop, functional hyperconnectivity between the thalamus/basal ganglia and the auditory-somatomotor network contributes to positive symptoms, whereas hypoconnectivity between the thalamus/basal ganglia and the middle frontal gyrus has been linked to cognitive deficits.^6^ Furthermore, negative symptom severity is associated with disturbed functional connectivity within the default-mode network.^9^ At the same time, deficits in cognitive domains such as processing speed and working memory have been reported to be linked to functional dysconnectivity within the salience, auditory, somatomotor and visual networks.^2^ Notably, cognitive deterioration is strongly related to impairments in social and occupational functioning^10,11^ and thus contributes to low recovery rates of patients with SSD.^12^ Hence, grounded in the dysconnection hypothesis of schizophrenia, the current state of research demonstrates that SSD is characterised by multiple dysconnectivity patterns across the whole brain that are closely intertwined with the main clinical symptoms of psychotic disorders, namely positive and negative symptoms, as well as cognitive deficits accompanied by impairments in daily life functioning.

To achieve further clinical improvements beyond treatment as usual, physical exercise interventions have been proposed as an add-on therapy in recent years. Large-scale evidence reveals beneficial effects of different types of physical exercise treatments in people with SSD on positive symptoms,^13,14^ negative symptoms,^13,14^ cognitive impairments^14,15^ and daily life functioning.^16^ These effects are thought to be driven by multiple structural and functional adaptations of the brain,^17^ but the underlying neural mechanisms are not fully understood. Preliminary evidence indicates that higher aerobic fitness, as an indicator of regular involvement in physical exercise, is associated with increased functional connectivity within the default-mode network in SSD.^18^ Concurrently, aerobic exercise may induce functional adaptations in the cortico-striato-pallido-thalamo-cortical loop and the default-mode network.^19^ However, the studies that reported these findings examined functional connectivity patterns within specific networks relevant to SSD without considering these patterns in relation to a healthy norm. Hence, previous evidence does not allow conclusions to be drawn about potential normalisation processes of functional connectivity following physical exercise treatment in SSD. To address this gap, spatially informed imaging transcriptomics is required. Concerning functional brain connectivity assessed by resting-state fMRI, Stoecklein et al^20–22^ have proposed the dysconnectivity index (DCI), which quantifies the deviation of functional connectivity from a healthy reference norm for each voxel in each subject and summarises these deviation scores as a whole-brain measure of functional dysconnectivity. The DCI can also be computed for specific brain regions and networks, in which case it is referred to as the specific DCI.

An imaging transcriptomics approach was proposed recently to provide further insight in the genetic underpinnings of macroscale neural alterations such as functional dysconnectivity.^23–25^ In this context, such a technique enables exploration of associations between functional dysconnectivity patterns in patients with SSD and general gene expression profiles in the human brain derived from the Allen Human Brain Atlas.^26^ Application of imaging transcriptomics has the potential to provide insights into the genetic origins of functional dysconnectivity in SSD, thereby offering a more sophisticated mechanistic understanding of the disorder.^23–25^

Here, combining the DCI with an imaging transcriptomics approach, we investigated connectome-based mechanisms that drive clinical improvements in schizophrenia and studied the genetic basis of changes in functional dysconnectivity, as a better understanding of such mechanisms could help to develop targeted therapies and guide future treatment decisions. First, we hypothesised that whole-brain and specific functional dysconnectivity would decrease after physical exercise treatment in individuals with SSD. Second, we assumed that reductions in functional dysconnectivity would be linked to improvements in total symptom severity, cognition and global levels of functioning. Third, we explored potential genetic underpinnings of reductions in functional dysconnectivity after physical exercise.

## Method

The current work is based on data from the Enhancing Schizophrenia Prevention and Recovery through Innovative Treatments (ESPRIT) C3 study coordinated by the Central Institute of Mental Health in Mannheim. The ESPRIT C3 study is a completed multicentre randomised controlled trial that explored the effects of aerobic endurance training (AET) compared with flexibility, strengthening and balance training (FBST) on several health outcomes in people with SSD. Details of the study and the main clinical results have been published elsewhere.^14,27^ The study is in line with the Declaration of Helsinki, and ethical approval was provided by the local ethics committee of the Ludwig Maximilian University Hospital (approval number: 706-15). With respect to the project at hand, only data that were acquired at the Department of Psychiatry and Psychotherapy and the Department of Radiology of the Ludwig Maximilian University Hospital in Munich were utilised. All participants provided written informed consent before their inclusion in the study, in accordance with ethical guidelines.

### Study design and sample

For the main ESPRIT C3 study, the intention-to-treat sample consisted of 180 individuals with SSD. Participants were randomly assigned to either AET or FSBT. Patients in both groups exercised up to three times per week, for between 40 and 50 min, for 6 months. In the AET group, participants cycled on a stationary bicycle ergometer at an individualised moderate exercise intensity determined via a stepwise lactate threshold test. Patients in the FSBT group performed various stretching, mobility, stability, balance and relaxation exercises. Details regarding the study design, randomisation process, blinding procedures, organisation of the exercise training and other relevant methodological considerations have been described elsewhere.^28,29^

Regarding the present work, only data from participants who underwent structural fMRI before and after 6 months of the intervention were considered. After rigorous quality control, a total of 23 people with SSD (21 had a diagnosis of schizophrenia and two had schizoaffective disorder) were considered in the final statistical analyses. We assumed that reductions in dysconnectivity could occur across different types of exercise. Thus, we did not include a non-exercise control group, as our primary focus was not on comparing exercise types or establishing specificity. The healthy reference sample for the dysconnectivity calculations comprised 200 individuals (120 female, 80 male), with an average age of 30.20 years (±3.19 years), using data from the Genomics Superstruct Project.^30^ This group was used solely for contextual comparison of dysconnectivity patterns. As only patients underwent the intervention, a repeated-measures analysis of variance including healthy controls was not suitable for testing our longitudinal hypotheses.

### MRI data acquisition

SSD patients were scanned with a 3T Siemens Magnetom Skyra MRI scanner (SIEMENS Healthineers AG, Erlangen, Germany) at the Department of Radiology of the Ludwig-Maximilians-University Hospital Munich. One 3D T1-weighted magnetisation-prepared rapid gradient echo sequence with an isotropic spatial resolution of 0.8 × 0.8 × 0.8 mm^3^ and two resting-state functional echo planar imaging sequences with a total duration of 12 min were acquired. Supplementary Table 1 available at https://doi.org/10.1192/bjo.2025.10892 displays the scanning parameters. The scanning protocol used for the Genomics Superstruct Project data closely resembled that of the current scanning protocol.

### Quality control and preprocessing

Structural and resting-state fMRI data were quality controlled on the basis of both visual inspection of images before and after preprocessing and automated quality control software MRIQC version 0.16.1 for Linux (Poldrack Lab, Stanford University, Stanford, CA, USA; https://mriqc.readthedocs.io).^31^ The temporal signal-to-noise ratio (tSNR) was calculated voxel-wise as the mean signal across time divided by the standard deviation across time. Mean tSNR values within the grey matter mask were then extracted to summarise data quality for each participant. All data obtained after preprocessing had a tSNR greater than 100 and a mean framewise displacement of less than 0.3 mm.

Structural MRI data were processed using FreeSurfer version 6.0 for Linux (Martinos Center for Biomedical Imaging, Massachusetts General Hospital, Boston, MA, USA; http://surfer.nmr.mgh.harvard.edu). Preprocessing of the resting-state fMRI was performed using fMRIPrep version 20.2.2 for Linux (Poldrack Lab, Stanford University, Stanford, CA, USA; https://fmriprep.org).^32^ The first five functional frames were removed, and the remaining frames were z-scored and smoothed with a 6.0 mm full width at half maximum Gaussian kernel. MCFLIRT (FSL version 5.0.9 for Linux) (Oxford Centre for Functional MRI of the Brain, University of Oxford, Oxford, UK; https://fsl.fmrib.ox.ac.uk) was applied for subsequent head motion correction using ICA-AROMA FSL version 5.0.9 for Linux (Oxford Centre for Functional MRI of the Brain, University of Oxford, Oxford, UK; fsl.fmrib.ox.ac.uk).^33^ Last, nuisance regression, band-pass filtering (0.01 to 0.08 Hz) and detrending were performed.

For the whole-brain DCI, blood oxygenation-level-dependent signals were extracted for each subject, and correlation matrices were calculated separately for the left hemisphere (3352 voxels) and right hemisphere (3316 voxels). These correlation matrices were then transformed using the Fisher Z-transformation. For the specific DCI, correlations were calculated between signals from predefined seed regions corresponding to networks or brain areas of interest and were likewise Fisher Z-transformed. Further methodological details are provided in the Supplementary Material.

### Calculation of the whole-brain DCI

The DCI was computed using an approach similar to those outlined in prior work by Stoecklein et al,^20^ as detailed in the Supplementary Material. In essence, connections deviating beyond a specific threshold from the distribution observed in the reference group were classified as ‘dysconnected’. The dysconnectivity count (DCC) for each patient was then aggregated within each hemisphere and normalised by the number of voxels in that hemisphere to yield the whole-brain DCI.

### Calculation of the DCI within specific brain regions and networks

In order to probe subtle changes in brain connectivity, we extracted pertinent brain voxels from both the reference group and schizophrenia patients. We examined these voxels across multiple networks, encompassing the visual, somatomotor, limbic, frontoparietal, default-mode, dorsal attention, salience and subcortical networks. In addition, we explored connectivity within specific brain regions of interest, such as the hippocampal formation and between the hippocampus and the prefrontal cortex, the thalamus and the middle frontal gyrus, and the thalamus and the somatomotor network. Following that, we calculated the DCC exclusively for each of these designated brain regions and networks, yielding a specific DCI that encapsulated these specific functional dysconnectivity patterns.

### Assessment of whole-brain DCC changes and clinical outcomes

The Global Assessment of Functioning (GAF) scale was administered to assess the general status of psychiatric symptoms and functioning.^34^ To cover total schizophrenia symptom severity, consisting of positive and negative symptoms, as well as general psychopathology, the Positive and Negative Syndrome Scale (PANSS)^35^ was utilised. To measure global cognitive performance, several cognitive tests that targeted different subdomains of cognition (e.g. short- and long-term memory, working memory, verbal fluency, sustained attention) were performed, and their scores were summarised to give a composite score (for details, see the Supplementary Material). All clinical measures were acquired at baseline before the start of the intervention and post-intervention after 6 months of physical exercise. The changes in scores between baseline and post-intervention were calculated in such a way that positive values represented an improvement and negative values represented a deterioration on the respective scales. The whole-brain DCC of each voxel was normalised across all participants and the score changes were computed by subtracting the DCC for each voxel at baseline from the respective DCC at follow-up. Hence, a positive DCC change score reflected a reduction in dysconnectivity of the corresponding voxel.

### Statistical data analysis

To examine whether whole-brain and specific functional dysconnectivity in people with SSD decreased after physical exercise treatment, we calculated 13 linear mixed effect models for repeated measures using the lme4 package^36^ in R version 4.2.2 (R Foundation for Statistical Computing, Vienna, Austria; r-project.org) using the lme4 package version 1.1-31 (Department of Statistics, University of Wisconsin–Madison, USA; cran.r-project.org/package=lme4). Session (baseline, 6 months), age, sex, chlorpromazine (CPZ) equivalents, number of achieved exercise sessions (*Trainings*_*i*_), group (AET, FSBT) and hemisphere (left, right) were included as fixed effects, whereas the whole-brain and specific DCIs served as dependent variables. A random intercept was included for each participant. The model could be expressed as follows:

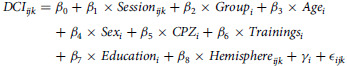




where *DCI*_*i*, *j*, *k*_ represents the observed DCI of the *i*th subject at the *j*th time point in the *k*th hemisphere; *β*_0_ reflects the intercept of the whole model; the *β*_*n*_ × *X*_*ijk*_ terms represent the values of fixed effects with their coefficients for the *i*th participant (*i* = 1, …, *n*) at the *j*th time point (*j* = 1, 2) in the *k*th hemisphere (*k* = 1, 2); *γ*_*i*_ captures the random intercept of the *i*th participant; and *ε*_*ijk*_ is the residual error term.

The *P*-values of the predictor *Session* were extracted and corrected for multiple testing across the 13 linear mixed effect models using the false discovery rate (FDR) method. In the case of significance (*q* < 0.05) after FDR correction, Tukey *post hoc* tests for the particular DCI outcome were performed to identify the direction and size of the effect, expressed as Cohen´s *d*, and the respective 95% confidence interval.

To investigate the relationship between changes in the DCC and clinical improvements, we conducted voxel-wise linear regression analyses using GAF, PANSS and cognitive composite score changes as dependent variables. Each model controlled for potential confounders, including age, sex and CPZ dose equivalents. For every voxel, we extracted the regression *β*-values (effect sizes) and corresponding *P*-values. To visualise the spatial distribution and direction of significant associations, we generated *β* maps thresholded at *P* < 0.05.

### Imaging transcriptomics approach

We first created DCC brain maps in the MNI standard space at 2 mm isotropic resolution, indicating the severity of dysconnectivity for each participant of each voxel at both time points. Using the glm module in Nilearn version 0.10.4 for Linux (INRIA Parietal Team, Saclay-Ⓘle-de-France, France; nilearn.github.io), we computed a voxel-wise analysis of variance for repeated measures including session (baseline, 6 months), age, sex, CPZ equivalents, number of achieved exercise sessions and group (AET, FSBT) as factors. We derived continuous statistical brain maps indicating the voxel-wise change of dysconnectivity from pre- to post-intervention across the whole sample. These maps were parcellated according to the Brainnetome atlas using the parcellate module from neuromaps (Neuromaps version 0.0.5 for Linux; McGill University, Montreal, Canada; netneurolab.github.io/neuromaps).^37^ Analogously, we parcellated the Allen Human Brain Atlas based on the Brainnetome atlas in MNI space at an isotropic resolution of 2 mm using the abagen toolbox (Abagen version 0.1.3 (2021) for Windows; McGill University, Montreal, Canada; abagen.readthedocs.io).^38^ On the basis of the resampled and parcellated statistical DCC brain map, we generated respective null models according to Burt et al^39^ to preserve spatial autocorrelation and computed Spearman correlations between DCC changes and gene expression data using the stats module from neuromaps.^37^ This resulted in a vector of correlation coefficients for each gene included in the Allen Human Brain Atlas, revealing the correlation between functional dysconnectivity changes in patients and gene-specific expression in the human brain across all regions defined in the Brainnetome atlas. To gain insight in the gene types that showed consistent associations with changes in functional dysconnectivity, we used this vector to perform gene set enrichment analysis on the basis of the corresponding gene-to-cell-type annotations provided by Lake et al^38^ using the gseapy toolbox (GSEAPy version 1.0.3 (2023) for Linux; Bioinformatics Center, University of Copenhagen, Copenhagen, Denmark; gseapy.readthedocs.io).^39^ For a detailed explanation of the general principles of the imaging transcriptomics approach, see Arnatkeviciute et al.^40^

## Results

### Patient characteristics

The majority of the final study sample was male, middle-aged, well-educated and in the post-acute phase of the disease. Moreover, most of the participants considered here were randomised to the FSBT exercise group, having on average completed 1.3 exercise sessions per week. The detailed characteristics of the study sample are summarised in Table 1.

**Table 1:** Sample characteristics

Characteristics	Patients with SSD *N* = 23^a^ *n* (%) or mean ± s.d.
Exercise group	
AET	9 (39.1)
FSBT	14 (60.9)
Sex	
Female	7 (30.4)
Male	16 (69.6)
Age, years	37.17 ± 10.38
PANSS at baseline	
Positive symptoms	11.17 ± 3.39
Negative symptoms	10.65 ± 3.31
Total symptoms	45.74 ± 9.48
GAF at baseline	64.57 ± 7.79
Chlorpromazine equivalents^b^	428.04 ± 243.76
Education, years	16.30 ± 4.16
Total number of training sessions	34.52 ± 15.86

AET, aerobic endurance training; FSBT, flexibility, strengthening and balance training; PANSS, Positive and Negative Syndrome Scale; GAF, Global Assessment of Functioning Scale.

a. The sample size refers to the number of participants that were considered in the final statistical data analysis (*N*, total sample size; *n*, sample size per category).

b. Chlorpromazine equivalents were computed according to the defined daily doses method.

### Changes in whole-brain and specific functional dysconnectivity after physical exercise

**Fig. 1:**
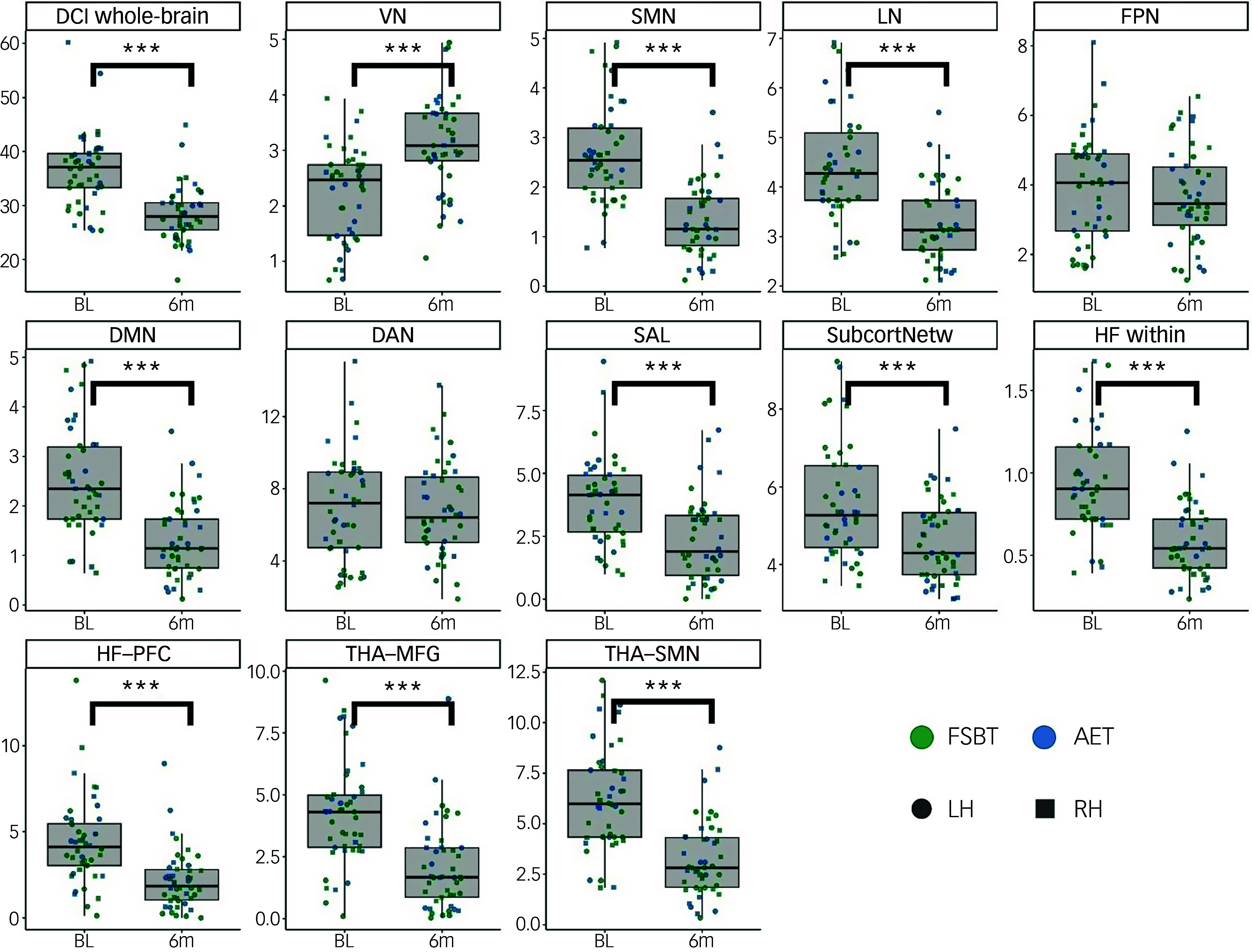
Changes in whole-brain and specific DCI from baseline to post intervention. The AET group is indicated in blue, the FSBT group in green. Circles reflect the left hemisphere, squares the right hemisphere. BL, baseline; 6m, post-intervention after 6 months of physical exercise; DCI, dysconnectivity index; VN; visual network; SMN, somatomotor network; LN, limbic network; FPN, frontoparietal network; DMN, default-mode network; DAN, dorsal attention network; SAL, salience network; SubcortNetw, subcortical network; HF, hippocampal formation; PFC, prefrontal cortex; THA, thalamus; MFG, middle frontal gyrus; FSBT, flexibility, strengthening, and balance training; AET, aerobic endurance training; LH, left hemisphere; RH, right hemisphere. ***P < 0.001.

The predictor *Time* was significant after FDR correction for the following whole-brain and specific DCI variants: whole brain (*q* < 0.001), visual network (*q* < 0.001), somatomotor network (*q* < 0.001), limbic network (*q* < 0.001), default-mode network (*q* < 0.001), salience network (*q* < 0.001), subcortical network (*q* < 0.001), thalamic-prefrontal connection (*q* < 0.001), thalamic-somatomotor connection (*q* < 0.001), hippocampal-prefrontal connection (*q* < 0.001) and connectivity within the hippocampal formation (*q* < 0.001). These effects indicated changes over time in the corresponding DCI values across both exercise groups. There was no significant change in the specific DCI for the frontoparietal and dorsal attention network. Tukey *post hoc* tests demonstrated significant reductions from baseline to post-intervention for the whole-brain DCI (*d* = −2.73, CI [−3.15, −2.31], *P*_Tukey_ <0.001) and the specific DCI of the somatomotor network (*d* = −2.52, CI [−2.94, −2.12], *P*_Tukey_<0.001), limbic network (*d* = −1.87, CI [−2.28, −1.45], *P*_Tukey_ <0.001), default-mode network (*d* = −2.19, CI [−2.61, −1.78], *P*_Tukey_ <0.001), salience network (*d* = −1.84, CI [−2.25, −1.42], *P*_Tukey_ <0.001), subcortical network (*d* = −1.18, CI [−1.59, −0.76], *P*_Tukey_ <0.001), thalamic-middle frontal connection (*d* = −1.47, CI [−1.89, −1.05], *P*_Tukey_ <0.001), thalamic-somatomotor connection (*d* = −2.25, CI [−2.67, −1.84], *P*_Tukey_ <0.001), hippocampal-prefrontal connection (*d* = −1.34, CI [−1.76, −0.92], *P*_Tukey_ <0.001) and connectivity within the hippocampal formation (*d* = −2.19, CI [−2.61, −1.78], *P*_Tukey_ <0.001). Increases in the DCI were observed for the visual network (*d* = 1.45, CI [1.03, 1.86], *P*_Tukey_ <0.001). Figure 1 illustrates the time effects on all computed DCI scores. The full test statistics of the other fixed effects are summarised in Supplementary Table 2.

### Brain-region- and network-specific correlations between reductions of whole-brain DCC and clinical improvements

**Fig. 2:**
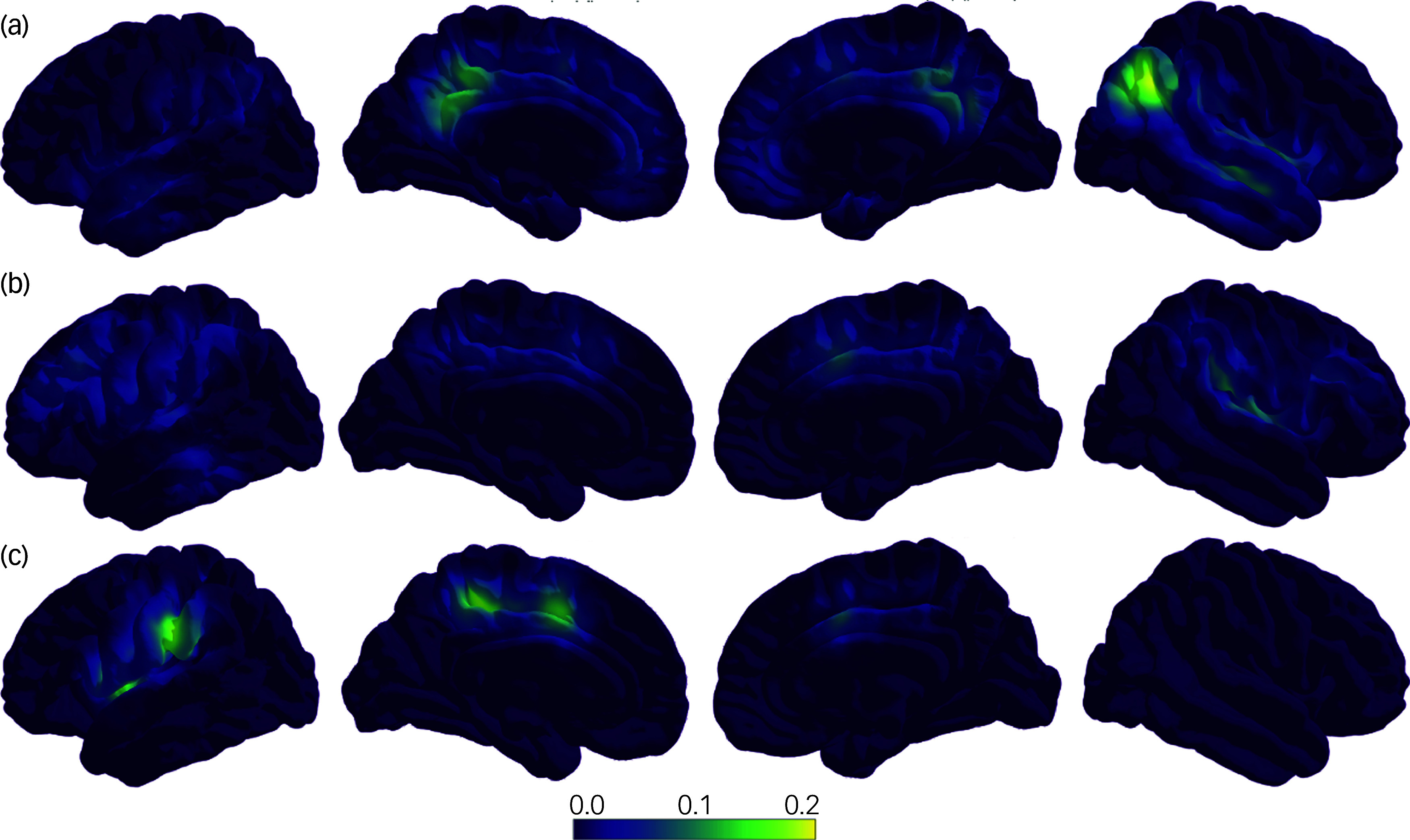
Correlations between DCC change and change in clinical measurements, illustrating, on a surface level, the voxel-wise linear regression analyses using GAF score, PANSS score and cognition changes as dependent variables to DCC change. (a) For GAF scores, a strong association was observed in the DMN across both hemispheres. The insular region and ACC forming the salience network in the right hemisphere also showed associations. (b) Changes in PANSS scores were primarily linked to the insular region in the right hemisphere, indicating involvement in the salience network. In addition, PANSS score was associated with the frontoparietal/somatomotor network. (c) Cognition was primarily associated with the left hemisphere, particularly in language areas such as Broca’s and Wernicke’s areas, as well as connecting tracts. Each model controlled for age, sex and chlorpromazine dose equivalents. For every voxel, we extracted the regression *β*-values (effect sizes) and corresponding *P*-values. To visualise the spatial distribution and direction of significant associations, we generated *β* maps thresholded at *P* < 0.05 The left hemisphere is displayed on the left side, whereas the right hemisphere is on the right. Yellow indicates a strong association between DCC and the respective clinical assessment; purple indicates no association. ACC, anterior cingulate cortex; DCC, dysconnectivity count; PANSS, Positive and Negative Syndrome Scale; GAF, Global Assessment of Functioning Scale.

As shown in Fig. 2(a), the strongest associations were between improvement in GAF and adaptations in DCC in the default-mode network, based on voxel-wise linear regression controlling for age, sex and CPZ dose equivalents. Reduced dysconnectivity in insular regions was associated with improvements in GAF and PANSS scores. Furthermore, dysconnectivity in somatomotor and frontoparietal networks was predominantly associated with improvements in PANSS score (Fig. 2(b)). Finally, improved cognition predominantly manifested through a reduction in dysconnectivity, notably observed in left hemisphere language regions such as Broca’s area (Fig. 2(c)).

### Association between changes in functional dysconnectivity and gene expression

**Fig. 3:**
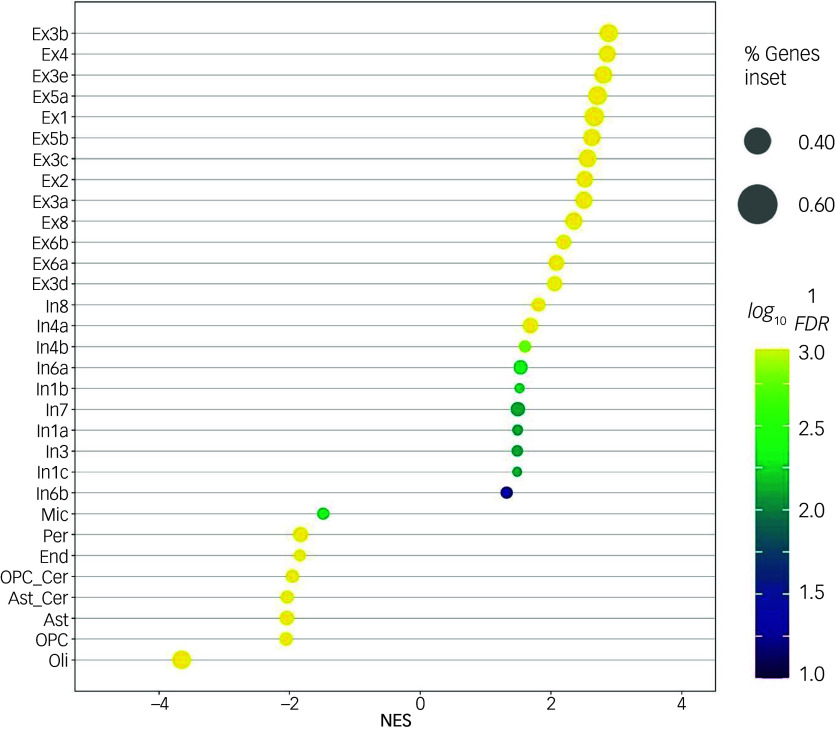
Results from the gene set enrichment analysis. The normalised enrichment score (NES) is shown on the *x*-axis; the cell types are displayed on the *y*-axis. The size of the dots indicates the proportion of genes in the gene set that showed an association with changes in functional dysconnectivity. The colours reflect the level of significance. Only significant gene sets are displayed (*q* < 0.05). Ex, excitatory neurons; In, inhibitory neurons; Mic, microglia; Per, pericytes; End, endothelial cells; OPC_Cer, cerebellar-specific oligodendrocyte precursor cells; Ast_Cer, cerebellar-specific astrocytes; Ast, astrocytes; OPC, oligodendrocyte precursor cells; Oli, oligodendrocytes; FDR, false discovery rate.

Figure 3 shows a summary of the results of the gene set enrichment analysis. Across brain regions, we found that reductions in functional dysconnectivity from baseline to post-intervention were linked to higher expression of genes typically enriched in oligodendrocytes, oligodendrocyte precursor cells (OPCs), astrocytes, endothelial cells, pericytes and microglia. By contrast, reductions in functional dysconnectivity from baseline to post-intervention were associated with lower expression of genes typically enriched in excitatory and inhibitory neurons.

## Discussion

In the current study, we explored the clinical relevance of potential decreases in functional dysconnectivity after physical exercise treatment in people with SSD and the respective genetic underpinnings, aiming to identify connectome-based mechanisms that drive clinical improvements.

We observed substantial reductions in the whole-brain DCI after physical exercise therapy among individuals with SSD. To our knowledge, this is the first time such a finding has been reported. Previous results in individuals with manifest SSD and in those at clinical high-risk for psychosis suggest adaptations of single functional connections induced by aerobic exercise interventions within the default-mode network or the cortico-striato-pallido-thalamo-cortical loop, or between hippocampal and occipital regions.^19,41,42^ These findings are in line with our current results, as they indicate that functional connectivity patterns in SSD can generally change in the context of a physical exercise intervention. However, direct comparisons with the current findings remain challenging owing to substantial differences in methodology. In particular, the distinctive approach of using the DCI in contrast to conventional measures of functional connectivity enabled us to obtain unique insights into individual-specific deviations in functional connectivity. In our case, reductions in functional dysconnectivity after physical exercise could be interpreted as normalisation processes of the individual brains of patients. Such inference would not be possible when only focusing on functional connectivity changes, as in previous studies.^19,41,42^ From a clinical standpoint, in contrast to previous research, our objective was to investigate individual alterations in functional dysconnectivity over time and under two distinct exercise interventions. However, previous studies primarily compared a moderate aerobic exercise intervention against either another form of exercise regimen^19,41^ or a waitlist control.^42^ Hence, they aimed to investigate potential adaptations in functional connectivity particularly induced by aerobic exercise interventions compared with a control condition. Here, we were interested in potential changes in functional dysconnectivity after physical exercise treatment without claiming that the substantial reductions we obtained were specifically induced by exercise. Consequently, the large decrease in functional dysconnectivity observed in our data may have resulted from a complex interaction of several factors the patients experienced in the course of their study participation, such as social, psychological, exercise or even placebo effects. In our data, both exercise interventions led to comparable reductions in dysconnectivity indices. This supports the view that different exercise modalities may similarly influence brain function in schizophrenia. However, we emphasise that the observed reductions in DCI may also have been influenced by non-specific factors such as routine treatment adjustments. Importantly, the main objective of our study was to evaluate the DCI as a potential neuroimaging-based marker of symptom change, rather than to isolate exercise-specific effects.

As we did not aim to identify the specific impact of exercise on functional dysconnectivity, the accumulation of these different effects may have led to the strong decreases in whole-brain functional dysconnectivity observed across time. Thus, the substantial reductions in whole-brain functional dysconnectivity observed in our data were not attributable solely to the effects of physical exercise but demonstrated that such interventions, with all their diverse impactful components, can be used to counteract functional brain dysconnectivity in SSD. This is of particular importance because SSD is seen as a disorder of dysconnectivity both on the theoretical^1^ and the empirical level.^3^

Beyond reductions in whole-brain functional dysconnectivity, we also found specific decreases in multiple networks and regions relevant to SSD, such as the default-mode network, salience network, somatomotor network, limbic network, subcortical network, prefrontal-thalamic-somatomotor pathway, hippocampal-prefrontal connections, and within the hippocampal formation. Multiple large-scale examinations have identified dysconnectivity patterns in these networks and regions as robust neural endophenotypes in SSD.^2,3^ Remarkably, our findings demonstrate that dysconnectivity in these areas can be mitigated in the context of an intervention such as physical exercise therapy. For the default-mode network and the prefrontal-thalamic-somatomotor pathway, our findings converged with our previous results on respective functional connectivity adaptations induced by aerobic exercise.^19^ However, our previous analyses did not detect changes in functional connectivity within the salience network, somatomotor network, limbic network, subcortical network, hippocampal-prefrontal connections or hippocampal formation in the aerobic exercise group compared with the flexibility, strengthening and balance group.^19^ Other findings have indicated effects of aerobic exercise on functional connectivity between the hippocampal formation and the occipital cortex.^41,42^ To conclude, the current results show that interventions such as physical exercise can be used to mitigate functional brain dysconnectivity in several brain networks and regions that have crucial roles in the pathophysiology of schizophrenia. Notably, we observed a substantial increase in functional dysconnectivity in the case of the visual network, whereas there was no change in the frontoparietal and dorsal attention networks. Although the mechanisms underlying this finding remain unclear, several speculative explanations may be considered. One possibility is that altered visual processing represents a compensatory response or plasticity effect related to physical activity, particularly given the known links between exercise and enhanced visual–spatial attention. Alternatively, visual network dysconnectivity might reflect subtle aggravation of symptoms tied to perceptual abnormalities, such as visual hallucinations, which have been associated with altered visual network function in schizophrenia. Prior studies have shown that increased functional connectivity in occipital regions may correspond with the emergence or exacerbation of positive symptoms in some patients.^43,44^ This indicates that functional dysconnectivity in SSD does not decrease consistently across the whole brain after physical exercise therapy, but the observed reductions in whole-brain functional dysconnectivity seem to be driven by certain networks and regions. The reasons for this need to be investigated in further large-scale interventional studies.

Regarding the clinical implications of changes in functional dysconnectivity, our findings suggested that reductions in functional dysconnectivity in the default-mode network were linked to improvements in global functioning. The default-mode network is generally associated with self-referential and interoceptive behaviour, theory of mind and social cognition.^45,46^ In the psychiatric context, disorder-related alterations within the default-mode network have been linked to negative symptom severity in people with SSD.^9^ Our previous analysis indicated that aerobic-exercise-induced increases in volume of the posterior cingulate gyrus, a central node of the default-mode network, were linked to an improvement in general disorder severity.^19^ These results reveal the broad and diverse functionality of the default-mode network in both healthy individuals and psychiatric patients. Our findings on the associations between modifications of dysconnectivity in the default-mode network and changes in global levels of functioning correspond to this notion. In sum, we propose that functional dysconnectivity within the default-mode network may reflect a promising neural target for future therapeutic approaches to improve the global level of functioning in people with SSD. Targeted treatment candidates may include brain stimulation approaches such as focused ultrasound or transcranial magnetic stimulation, which both have the potential to modulate functional connectivity.^47,48^

Our findings further indicated that a reduction in functional dysconnectivity in insular regions and the somatomotor network was associated with ameliorations in total symptom severity. Alterations of the insula in SSD have been associated with impaired ability of patients to process representations of themselves, leading to a disturbed discrimination between self-generated and external information.^49^ As the latter is thought to contribute to two main symptoms observed in people with SSD, namely hallucinations and delusions, our current findings on the relation between reductions in insular dysconnectivity and improvements in clinical symptoms seem plausible. With respect to the functional connectivity of the somatomotor network, previous evidence also suggests associations with different symptom domains of SSD.^50,51^ Hence, we conclude that reducing dysconnectivity in the insula and the somatomotor network through targeted therapeutic interventions such as the aforementioned brain stimulation techniques^50,52^ may represent a promising strategy to achieve amelioration of total symptom severity in patients with SSD.

Last, a decrease in functional dysconnectivity in language regions on the left hemisphere, such as Broca’s area, was related to cognitive improvements. To our knowledge, this is the first time such a finding has been described in people with SSD. In general, parts of Broca’s area are components of a broader left-hemispheric language network involved in language processing, whereas other parts contribute to wider cognitive functions such as explicit and working memory.^29,53^ Given that the cognitive composite score used in the current study comprised several cognitive tests that required language and memory functions (Supplementary Material), the observed link between dysconnectivity changes in Broca’s area and cognitive adaptations seems reasonable. Consequently, achieving decreases in functional dysconnectivity in Broca’s area using brain stimulation approaches may help to increase cognitive benefits in future treatments for people with SSD.

Regarding the genetic underpinnings of reductions of functional dysconnectivity after physical exercise in SSD, we observed that these reductions were most strongly associated with higher expression of genes typically enriched in oligodendrocytes and OPCs. In other words, in brain regions that showed stronger reductions in functional dysconnectivity, the expression of genes related to oligodendrocytes and OPCs was generally more pronounced. This is the first time such a finding has been reported. However, a recent theory of the aetiology of cognitive deficits in SSD proposed that erroneous maturation of OPCs into oligodendrocytes leads to impaired myelination and disturbed micro- and macroscale connectivity, manifesting in global cognitive deteriorations.^54^ This theory is based on a wide range of findings supporting the involvement of OPCs and oligodendrocytes in the pathophysiology of schizophrenia. For instance, among other cell types, OPCs and oligodendrocytes have been found to be partly enriched in genes linked to SSD,^55^ whereas several single genes with prominent roles in the functioning of OPCs and oligodendrocytes have been implicated in reduced structural connectivity in SSD.^54^ In addition, several stereology studies have revealed reductions in numbers of oligodendrocytes in people with SSD compared with controls.^56–58^ Our current results can be embedded in this line of evidence, as they indicate that reductions of functional dysconnectivity in SSD mainly occur in brain regions in which the expression of genes related to OPCs and oligodendrocytes is higher. This may point towards a remyelination process that may have occurred in the context of the physical exercise intervention, although such inferences should be interpreted with caution given the limitations of an imaging transcriptomics approach.

As outlined previously, we did not aim to examine the explicit effects of physical exercise on functional dysconnectivity. However, from a mechanistic perspective, the observed reductions in whole-brain and specific functional dysconnectivity in SSD and the clinical implications described above seem plausible in the context of physical exercise interventions. In general, exercise engagement is thought to have widespread neurobiological effects that can improve whole-brain and specific brain connectivity. For instance, exercise can stimulate expression of growth factors such as brain-derived neurotrophic factor, which in turn enhances synaptogenesis, synaptic transmission and dendritic spine density.^17,59,60^ Moreover, exercise can facilitate increased expression of genes involved in cell growth, synaptic trafficking or signal transduction, leading to general improvements in synaptic plasticity.^17^ Engaging in exercise is further thought to affect several core neurotransmitter systems, including the serotonergic, dopaminergic, glutamatergic, acetylcholinergic and norepinephrinergic pathways, which are essential for efficient neural information processing.^17^ Last, evidence from animal models indicates that exercise can support myelination and gliogenesis,^17^ consistent with our current findings on OPCs and oligodendrocytes. In general, these neural mechanisms facilitate effective signal transfer between different brain regions. Consequently, the whole-brain and specific reductions in functional dysconnectivity after physical exercise therapy in patients with SSD observed in the current study may have resulted from such multifaceted neural mechanisms at the micro level.

Despite these promising findings, our study had several important limitations that with implications for future research. First, only 22 patients with SSD could be included in the final analysis, although the main ESPRIT C3 study enrolled a total of 180 participants. This was mainly because participation in the MRI sessions was voluntary and was not part of the main study rationale, which had a clinical focus. Thus, the study population of people with SSD, on which the current analysis was based, was at a post-acute and stable phase of the disease; this prevented our findings from being generalisable to a broader spectrum of patients with SSD. In particular, the very large effect sizes observed here regarding dysconnectivity reductions may have been sample-specific and would probably not be obtained in more representative patient samples. Therefore, our results require replication in an independent and larger sample of people with SSD assessed using a comparable interventional design. Second, owing to the lack of a control group of healthy individuals, we could not control for technical scanner variance and natural fluctuations inf functional dysconnectivity patterns that may have occurred between measurement time points. However, we expected technical variability to be relatively low, given the rigorous quality control procedures applied. Any remaining technical variability should not have induced a systematic bias, as the exact time periods at which the 22 patients participated in the study were randomly distributed across multiple years of study implementation. Furthermore, we assumed that potential natural fluctuations in functional dysconnectivity that may be present even in healthy cohorts would not explain the substantial and systematic effects obtained in this analysis. Nonetheless, future studies of this type should include a longitudinal cohort of healthy controls, allowing technical variability and natural changes in functional dysconnectivity to be systematically accounted for. Third, the voxel-wise correlations between adaptations in functional dysconnectivity and clinical changes reflected descriptive associations without systematic significance testing and correction for multiple comparisons. We decided to follow a more liberal statistical approach as the first step, to generate hypotheses for subsequent studies. Hence, future hypothesis-driven research approaches in this area should evaluate whether the present associations between clinical changes and adaptations in functional dysconnectivity in the default-mode network, insula, somatomotor network and Broca’s area are reproducible using more rigorous statistical methods. If this is the case, future therapeutic approaches could aim to reduce functional dysconnectivity in the default-mode network, insula, somatomotor network, and Broca’s area to achieve multifaceted improvements on the clinical level.

## Supporting information

Wunderlich et al. supplementary material Wunderlich et al. supplementary material

## Data Availability

The data that support the findings of this study are available on request from the corresponding author, S.W. The data are not publicly available owing to their containing information that could compromise the privacy of research participants.

## Supplementary material

The supplementary material is available online at https://doi.org/10.1192/bjo.2025.10892
